# A Hydrophilic Sulfated Resveratrol Derivative for Topical Application: Sensitization and Anti-Allergic Potential

**DOI:** 10.3390/molecules28073158

**Published:** 2023-04-02

**Authors:** Ana Jesus, Ana I. Sebastião, Gonçalo Brites, Marta Correia-da-Silva, Honorina Cidade, Maria T. Cruz, Emília Sousa, Isabel F. Almeida

**Affiliations:** 1 UCIBIO—Applied Molecular Biosciences Unit, MedTech, Laboratory of Pharmaceutical Technology, Department of Drug Sciences, Faculty of Pharmacy, University of Porto, 4050-313 Porto, Portugal; 2 Associate Laboratory i4HB, Institute for Health and Bioeconomy, Faculty of Pharmacy, University of Porto, 4050-313 Porto, Portugal; 3 Faculty of Pharmacy, University of Coimbra, 3004-531 Coimbra, Portugal; 4 Center for Neurosciences and Cell Biology, 3004-504 Coimbra, Portugal; 5 Laboratory of Pharmaceutical and Organic Chemistry, Department of Chemical Sciences, Faculty of Pharmacy, University of Porto, 4050-313 Porto, Portugal; 6 CIIMAR—Interdisciplinar Centre of Marine and Environmental Research, Avenida General Norton de Matos, S/N, 4450-208 Matosinhos, Portugal

**Keywords:** resveratrol, sensitization potential, anti-allergic potential, safety profile, cosmetics

## Abstract

Resveratrol (RSV), a naturally occurring metabolite, is widely used in skincare products, but its hydrophobicity impairs its own incorporation into cosmetic formulations. RSV-GS is a synthetic hydrophilic sulfated glycosylated derivative inspired by marine natural products that present a lower cytotoxicity than RSV while exhibiting similar levels of bioactivity. Herein, we predict the skin sensitization potential of this new compound using an in vitro approach based on the OECD 442E guideline. Furthermore, the anti-allergic potential of RSV-GS was also disclosed. The monocyte THP-1 cell line was stimulated with RSV and RSV-GS in the presence or absence of the extreme skin allergen 1-fluoro-2,4-dinitrobenzene (DNFB). The results demonstrated that RSV-GS alone (500 µM) evoked a relative fluorescence index (RFI) lower than the thresholds established by the OECD guideline for CD54 (200%) and CD86 (150%), indicating the absence of a skin sensitization potential. Interestingly, in the presence of the skin allergen DNFB, RSV-GS exhibited the ability to rescue the DNFB-induced maturation of THP-1 cells, with RFI values lower than those for RSV, suggesting the potential of RSV-GS to mitigate skin sensitization evoked by allergens and, consequently, allergic contact dermatitis. These results open new avenues for the use of RSV-GS as a safe and anti-allergic active cosmetic ingredient.

## 1. Introduction

The skin is the largest organ in the human body, and it protects against chemical and physical injuries, maintaining skin homeostasis [1]. The skin also serves as a sensory organ and provides innate and adaptive immune responses. Several skin modifications have been described, mostly caused by UV radiation, external microorganisms, such as bacteria, fungi, or virus exposure, contact with chemicals, or genetic factors [1,2]. Other skin disturbances can also be driven by immune system issues, illnesses, and normal aging [2,3]. Currently, over 4000 substances have been classified as skin allergens, with some of them found in a wide range of consumer products such as cosmetics, cleaning agents, and perfumes [4,5,6,7,8]. Indeed, skin sensitization has been identified as a critical adverse effect of a wide range of chemicals, and the related disease, allergic contact dermatitis (ACD), can severely impact one’s quality of life [7]. ACD is a common skin disease that is steadily growing in prevalence; it is expected that 20% of the population in Western Europe is sensitized to one or more chemicals from their environmental setting [9]. This pathology has a considerable social and economic influence and is one of the most frequent presentations of work-related skin disorders. From a pathophysiological point of view, ACD includes the activation of both the innate and adaptative sides of the immune system and is divided into two distinct phases: the initial sensitization phase, where skin interacts with the sensitizing substance for the first time, driving the development of effector T cells, and the elicitation or challenge phase, which occurs after a second and successive contact attempts with the causative agent, where the symptoms develop [7]. Skin allergens are highly reactive, first reacting with endogenous proteins, making them immunogenic. These proteins conjugate and induce stress responses and xenoinflammation through the release of danger signals, such as the production of reactive oxygen species (ROS), uric acid, hyaluronic acid fragments, and adenosine triphosphate (ATP) and adenosine di-phosphate (ADP) by keratinocyte skin cells [7,10]. These danger signals trigger intracellular signaling pathways in antigen-presenting cells, such as Langerhans cells and dendritic cells (DCs), leading to their maturation [10]. Then, DCs process the protein conjugates and then migrate to the draining lymph nodes, where they prime naive T lymphocytes. T cells become activated and expand into allergen-specific effector T cells that disseminate systemically and elicit a strong inflammatory reaction upon later contact with the same chemical [10,11,12]. For preliminary studies, the Guidance Document on the Reporting of Defined Approaches and Individual Information Sources to be Used within Integrated Approaches to Testing and Assessment (IATA) for Skin Sensitisation (2012) described some physicochemical properties and in silico approaches to predict skin sensitization [13]. Furthermore, there are in vitro mechanistic-based assays available to assess the skin sensitization of new chemicals that are recommended by the European Union Reference Laboratory for Alternatives to Animal Testing and approved by the Organization for Economic Co-operation and Development (OECD). For instance, guideline 442E states that in vitro skin sensitization assays addressing the adverse outcome pathway key event on activation of dendritic cells are focused on the activation of Langerhans cells, which will culminate with their maturation and migration toward lymph nodes. [14].Specifically, The *h*-CLAT approach assesses alterations in the expression of cellular surface markers related to the maturation of dendritic cells (CD86 and CD54), and the THP-1 cell line has been used as a DC surrogate for skin sensitizing hazards [14]. In addition, other methods could be used for the assessment of skin sensitization, namely, the U937 cell line activation test (U-SENS^™^), the interleukin-8 reporter gene assay (IL-8 Luc Assay), and genomic allergen rapid detection (GARD^™^) [14]. 

The recent advances in the knowledge of the cellular and molecular mechanisms behind skin sensitization have opened the way for a better understanding of the complexity of ACD, concomitantly shedding light on the development of new preventive strategies. Indeed, the discovery of new compounds that are able to inhibit the allergic potential of new chemicals is of utmost importance. In the permanent search for new molecules, natural products have been widely explored for their diverse biological activities [15,16]. Flavonoids are a class of excellence due to their abundance in nature, in cereals, seeds, fruits, vegetables, and tea leaves [15]. Chalcone scaffold is widely found in terrestrial species, and is considered the main precursor of the biosynthesis of many flavonoids and polyphenolic compounds [17]. Since ever, flavonoid and polyphenolic compounds, including chalcones and stilbenes, have demonstrated their bioactive potential and diverse biological activities, such as anti-aging [18,19], anti-inflammatory [20,21], anti-microbial [22], anti-oxidant [20,21], and anti-tumor [23] activities. Resveratrol (RSV) is a naturally occurring stilbene produced by several plant species, which protects them from the attack of microorganisms and the noxious effects of UV radiation [24,25]. Its glycosylated derivative, 3-*O*-β-glucoside (RSV-G), is the most prevalent form found in nature [26]. Natural bioactive compounds from terrestrial and marine sources, either as extracts or inspiration for the synthesis of new molecules, are a mainstay for the discovery of new active compounds, including in the cosmetic field [27,28]. Nature mostly employs sulfation of endogenous and external substances to minimize any possible harm [29,30]. The sulfation reaction is thought to be a detoxification mechanism that enables easier removal from the body [29,30,31,32,33]. The increasing relevance of natural sulfated compounds as modulators of a variety of processes, both physiological and pathological, has guided the creation of non-natural sulfated scaffolds [31]. Flavonoids are a group of natural products that are widely reported for being sulfated [29,30], inspiring the synthesis of sulfated compounds [31,34,35]; however, this molecular strategy has been applied to other classes of compounds, including stilbenes and other polyphenols [35,36]. Resveratrol metabolism is influenced by sulfation reactions. Concretely, resveratrol is internalized into the enterocyte and then sulfated and glycosylated by sulfotransferase and glucuronidase enzymes [37,38]. This specific molecular modification could overcome the solubility obstacle of bioactive compounds with high hydrophobicity such as RSV. In this work, an initial in silico prediction of the reactive points of the new compound RSV-GS compared with RSV was performed. Aiming at the confirmation of the preliminary results, the sensitization potential and anti-allergic activity of RSV-GS were compared to RSV, highlighting and reinforcing the potential of this sulfated derivative already reported by our group [36], in order to be used in topical formulations.

## 2. Results and Discussion

### 2.1. Comparison of Reactive Points Using in Silico Software ADMETLab 2.0

In order to elucidate and predict the reactive points associated with the chemical structures of the compounds RSV and RSV-GS, an in silico study was performed regarding the characteristics related to the sensitization potential (Table 1), using the software ADMETlab 2.0 (https://admetmesh.scbdd.com/, accessed on 2 February 2023). RSV was also studied, and the results achieved were compared with those obtained with the RSV-GS. The “skin sensitization rule” was used to detect certain reactive points in the chemical structure of the compounds that were able to cause skin sensitization reactions. The higher the number of alerts exhibited, the higher the probability that a compound will induce skin sensitization [39].

Alerts detected for RSV (four alerts) and RSV-GS (three alerts) showed the probability of compounds evoking in vitro and in vivo skin sensitization reactions. Through the chemical structures drawn in Table 1 and highlighting the reactive points that were able to interact with the skin´s constituents, it is possible to observe that the two free aromatic hydroxyl groups, existing in the RSV structure, are not present as reactive points on the RSV-GS´ chemical structure. This suggests that the molecular modification performed, the sulfation of phenol groups, diminishes the probability that this derivative will induce sensitization reactions, contributing to the amelioration of the RSV-GS safety profile relative to RSV. Aiming to further explore the skin reactivity of these compounds, an in vitro human cell line activation test (*h*-CLAT) recommended for the 442E OECD guideline for skin sensitization evaluation was performed.

### 2.2. Skin Sensitization Potential of RSV and RSV-GS in THP-1 Cells 

**Figure 1:**
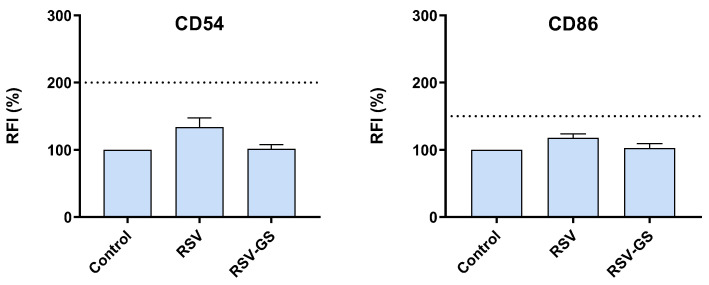
Skin sensitization potential of RSV and RSV-GS compounds. The relative fluorescence intensity (RFI) of CD54 and CD86 expression was measured. Bar graph presents the mean ± standard error of mean (SEM) of at least three independent experiments. Dashed line indicates the established thresholds for CD54 (200%) and CD86 (150%) markers. The comparisons were performed using one-way ANOVA analysis, with Dunnett’s multiple comparison post-test, where *p* < 0.05 was considered significant compared with the control.

The need for non-animal alternatives has been acknowledged for screening the threat of chemicals to human health in general. It has become especially pressing for cosmetic ingredients due to the European Cosmetics Regulation, which advocates a full ban on animal testing. Fortunately, proven alternatives for some endpoints, such as skin sensitization reactions, are currently available to be performed during new product development. So far, OECD has recommended four test methods, including the human cell line activation test (*h*-CLAT) [14]. All these assays are based on the adverse outcome pathway for skin sensitization, which includes four key events [7]. The first key event (KE1) is associated with the covalent bond between the hapten and skin proteins, where the electrophilic properties of the hapten facilitate the initiation of the process, permeating the skin layers and connecting to the skin proteins [7]. After that, the keratinocyte cells (KE2) will be activated, causing an increase in the inflammatory cytokine production, accompanied by the activation of Langerhans cells, which will culminate in the migration and maturation of those cells (KE3) [7]. KE1-3 is associated with molecular and cellular-level events. The last key event (KE4) consists of the presentation of the allergen to the T lymphocytes by mature Langerhans cells, ensuing the priming and activation of T lymphocytes that are specific for the allergen [7]. This will cause an inflammatory reaction, when the same allergen interacts with the skin on subsequent occurrences [7]. Indeed, the *h*-CLAT proved to accurately anticipate sensitizers that were extreme, strong, moderate, or weak, according to their categorization in the local lymph node assay (LLNA) [40]. For this reason, this alternative assay was employed herein to ensure the safety profile of the compounds. Following the OECD recommendations for skin sensitization evaluation, the up-regulation of the co-stimulatory molecules, CD54 and CD86, in THP-1 cells was used to exclude the molecules’ ability to cause skin sensitization (Figure 1). Regarding the skin sensitization’s potential, compounds RSV and RSV-GS showed lower RFI (%) values than the thresholds (CD54: 200% and CD86: 150%) established by the OECD guideline In Vitro Skin Sensitization: human cell line activation test (*h*-CLAT) [14]. Therefore, both compounds can be classified as negatives for sensitizers; thus, the non-sensitizers label could be applied for RSV and RSV-GS. Comparing RSV and RSV-GS, RSV-GS exhibited lower RFI (%) values than RSV for CD54 and CD86 markers. Concretely, for RSV-GS, the RFI (%) values for CD54 and CD86 markers were 101.3% and 102.6%, and for RSV they were 133.8% and 117.8%, respectively. These results suggest that the molecular modification performed ameliorates the safety profile, increasing the gap between the RSV (CD54: 133.8% and CD86: 117.8%) and RSV-GS (CD54: 101.3% and CD86: 102.6%).

### 2.3. Anti-Allergic Potential of RSV and RSV-GS in THP-1 Cells

**Figure 2:**
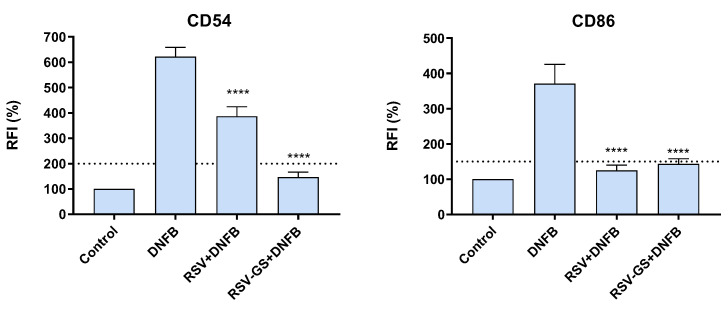
Anti-allergic activity of RSV and RVS-GS toward the maturation of THP-1 cells induced by the allergen DNFB. The relative fluorescence intensity (RFI) of CD54 and CD86 expression was measured. Bar graph presents the mean ± SEM of at least three independent experiments. Dashed line indicates the established thresholds for CD54 (200%) and CD86 (150%) markers. The comparisons were performed using one-way ANOVA analysis, with Dunnett’s multiple comparison post-test, where *p* < 0.05 was considered significant compared with the DNFB. **** *p* < 0.0001: significantly different compared to the DNFB.

The ability of the compounds to mitigate skin sensitization and, consequently, ACD was further examined using the potent allergen 1-fluoro-2,4-dinitrobenzene (DNFB). As expected, DNFB, which is a strong skin sensitizer, triggered THP-1 cells maturation, as assessed by the up-regulation of CD54 and CD86 markers. Interestingly, both chemicals suppressed the allergen-induced THP-1 cell maturation (Figure 2). In the evaluation of the sensitization potential of the chemicals (Section 2.2.), both RSV and RSV-GS demonstrated a favorable safety profile, with RSV-GS evoking lower values of RFI. The introduction of a sulfated glycosylated group to RSV was also found to be beneficial in this assay, with RSV-GS showing a higher inhibitory effect on DNFB-induced cell maturation relative to RSV. More specifically, the RFI (%) values for CD54 and CD86 membrane cell markers were 146.1% ± 31.81% and 143.7% ± 43.07% for RSV-GS and 386.0% ± 34.0% and 125.3% ± 39.54% for RSV, respectively. 

Taken together, these findings demonstrate that none of the evaluated compounds caused skin sensitization, supporting their safety profile for use in cosmetic and skincare applications. Furthermore, these compounds inhibit the THP-1 maturation induced by the extreme allergen DNFB, emphasizing the compounds’ anti-allergic properties. It is noteworthy to mention that RSV-GS demonstrated a safer profile than the original compound RSV. Indeed, in both assays performed for the evaluation of the skin sensitization potential and effectiveness in the inhibition of DNFB-maturation of THP-1 cells, the RFI values of RSV-GS were always below the thresholds of the maturation markers used (CD54: 200% and CD86: 150%), reinforcing its safe characteristics when compared to RSV. Moreover, the fact that RSV-GS was below the threshold defined by the OECD guideline, even in the presence of an allergen, DNFB, could extrapolate to the future, preventing and minimizing the action of this sulfated compound in the presence of other substances with allergic characteristics, thus eliminating the harmful effects that the allergens could have after contact with skin. Despite the results herein obtained regarding the skin sensitization potential of RSV and RSV-GS, some reports describe the sensitization and co-sensitization potential of RSV, particularly in the presence of other sensitizer substances [41,42]. For instance, a 39-year-old man was prescribed with an emollient cream containing RSV to treat 6-month erythematous dermatitis [41]. After two weeks of cream application, the erythema spread and became exudative, so patch tests were performed to understand the trigger of that reaction. The diagnosis was ACD due to pentylene glycol sensitization, with co-sensitization with RSV [41]. The results of sensitization due to RSV were doubtful; however, the risk of contact allergy was not discharged due to the probable formation of electrophilic oxidation products of RSV [41]. Interestingly, this is not the only case stated in the literature that associates ACD with RSV [42]. Degraeuwe et al. also confirmed the contact allergy to an RSV-containing cosmetic cream in a 69-year-old woman, which resolved seven days after she stopped using the cream [42]. RSV was previously tested using one of the alternative tests that are mentioned in guideline 442E of the OECD guidelines [43]. Due to stability issues of RSV, the assay was not fully performed, so the sensitization potential of this compound remains unknown [43]. The results obtained herein, through an in vitro method approved by the OECD and described in the 442E guideline, disclose for the first time the skin sensitization potential and inhibitory potential toward the DNFB allergen-induced maturation in THP-1 cells RSV and RSV-GS compounds. This insight points out relevant information envisioning the future incorporation of these compounds in cosmetic products. Additionally, other studies supplement the appealing characteristics of RSV-GS for cosmetic purposes. The synthetically sulfated glycosylated derivative of RSV (RSV-GS) was classified as “very soluble” (>1000 mg/mL of ultra-pure water), 1000 times more soluble than RSV and the glycosylated derivative (RSV-G) (<1 mg/mL) [36]. Regarding its cytotoxicity, RSV-GS is non-cytotoxic to the HaCaT cell line up to 5000 µM [36]. Contrary to what was verified with RSV-GS, RSV caused a considerable reduction in the HaCaT cell viability at 100 µM, and RSV g at 500 µM [36]. Metabolic activity and ATP levels were also examined to understand whether RSV-GS prevented the peroxide- and menadione-induced cytotoxicity, and a decrease in metabolic activity at concentrations between 500 µM and 100 µM of RSV-GS was observed; however, there were no alterations in the ATP levels at concentrations above 5000 µM [36]. Finally, the levels of sirtuin 1 (SIRT1), which possess a critical role in the aging process, were assessed, and it was noted that SIRT-1 levels increased after treatment with RSV-GS at a concentration of 500 µM [36]. 

## 3. Materials and Methods

### 3.1. Materials

RSV-GS was synthetized in the Laboratory of Organic and Pharmaceutical Chemistry of the Faculty of Pharmacy of the University of Porto, according to previously procedures [36]. Resveratrol (RSV) and the allergen 1-fluoro-2,4-dinitrobenzene (DNFB) were purchased from Sigma-Aldrich Chemical Co. (St. Louis, MO, USA). The antibodies used for stained conditions were anti-CD86 (clone IT2.2, reference 305414) and anti-CD54 (clone HA58, reference 353111), both of which were purchased from Biolegend (San Diego, CA, USA)

### 3.2. Software Used for the Prediction of Sensitization Potential and Reactive Points of Chemicals

ADMETlab 2.0 software (https://admetmesh.scbdd.com/, accessed on 2 February 2023) was used for the in silico studies of compounds RSV and RSV-GS regarding skin sensitization potential and reactive points in chemical structure.

### 3.3. Inhibition of THP-1 Maturation Profile Induced by the Strong Allergen DNFB

In compliance with OECD guidelines, the human monocyte THP-1 cell line has been employed as a dendritic cell (DC) surrogate for the skin sensitizing hazard. Adapting the OECD test guideline No. 442E, cell concentration was adjusted to 0.5 × 10^6^ cells/mL and stabilized overnight at 37 °C, with 5% of CO_2_. Then, 1.5 mL of cell culture was plated in each well of a 12-well plate (MW-12) and the chemicals RSV and RSV-GS were applied at the final non-cytotoxic concentrations of 50 µM and 500 µM, respectively. For compounds RSV and RSV-GS, the concentrations were selected based on viability assays that had already been performed and reported in the literature [36]. After a pre-incubation period of cells with the compounds (1 h at 37 °C with 5% of CO_2_), a solution of 8 mM DNFB in DMSO (at the final DMSO % in the cell incubation of 0.1) was added (final concentration of 8 µM) and cells were further incubated for 24 h, at 37 °C, with 5% of CO_2_. The cell control, cells with 0.1% DMSO, was incubated in the same conditions of the treated cells. Then, cells were centrifuged at 300 g for 5 min. The supernatant was rejected, and cell pellet was washed with 1 mL of PBS containing 1% FBS and centrifuged in the same conditions as the previous mentioned. After discarding the supernatant, the pellet was further resuspended in 1 mL of PBS/1% FBS and 200 µL was collected for two eppendorfs: 100 µL for the unstained (UNST) and 100 µL for the stained (CD5486) conditions. For each stained condition, 3 µL of anti-CD54 and 3 µL of anti-CD86 were added and incubated for 30 min, at 4 °C, and were protected by the light. After that, cells were washed with solution of PBS/1% FBS and centrifuged. Then, the supernatant was collected, and the cell pellet was resuspended in 100 µL of PBS containing 1% FBS, following analysis through flow cytometry technique, using the BD Accuri™ C6 cytometer (San Jose, CA, USA). 

### 3.4. Flow Cytometry Analysis

The CD86 and CD54 levels were analyzed by flow cytometry with the acquisition channel FL1 and FL4. The relative fluorescence intensity (RFI) of CD86 and CD54 markers for the positive control cells and chemical-treated cells, based on the geometric mean fluorescence intensity (MFI), was calculated according to the following equation:

(1) $$\mathrm{RFI}=\frac{\mathrm{MFI}\;\mathrm{of}\;\mathrm{chemical}\;\mathrm{treated}\;\mathrm{cells}-\mathrm{MFI}\;\mathrm{of}\;\mathrm{chemical}\;\mathrm{treated}\;\mathrm{unstained}\;\mathrm{cells}}{\mathrm{MFI}\;\mathrm{of}\;\mathrm{control}\;\mathrm{treated}\;\mathrm{cells}-\mathrm{MFI}\;\mathrm{of}\;\mathrm{control}\;\mathrm{unstained}\;\mathrm{cells}\;}\times 100$$

The results of at least three independent experiments were represented as a percentage of the control RFI, which was assumed to be 100%. The samples are designated as skin sensitizers if the RFI (%) of CD54 and CD86 is equal to or more than 200% and 150%, respectively.

### 3.5. Statistical Analysis

The statistical analysis was performed with GraphPad Prism8 for Windows (GraphPad Software, San Diego, CA, USA; www.graphpad.com, accessed on 25 February 2023). The results were expressed as mean ± standard error of the mean (SEM) of at least three independent experiments. For the results obtained in THP-1 maturation assay, the comparisons were performed using one-way ANOVA analysis, with Dunnett’s multiple comparison post-test, where *p* < 0.05 was considered significant. The statistical significance levels were: **** *p* < 0.0001.

## 4. Conclusions

Molecular modification is a strategy often used in medicinal chemistry that can be useful to overcome the drawbacks of active cosmetic ingredients. Low water solubility could be troublesome since the majority of cosmetic formulations have an aqueous external phase. RSV is a bioactive compound that is vastly reported in skincare applications. This compound has, however, poor aqueous solubility, which limits the maximum concentration that can be used in topical formulations. RSV-GS is a synthetic derivative of RSV, which was already reported to exhibit the potential to become an active ingredient for cosmetic applications. RSV-GS showed an improved solubility, an important hydrophilic profile, and a lower cytotoxicity in an epidermal keratinocyte cell line. The evaluation of the safety profile of new derivatives is mandatory, highlighting the need for combining a set of properties. One main concern is the potential of the new ingredients to evoke skin sensitization. Furthermore, the identification of new molecules that are able to prevent the development of skin sensitization is of utmost importance since there are more than four thousand identified allergens in diverse products of daily use. To assess the safety profile of RSV-GS, the sensitization and anti-allergic activities were evaluated. A preliminary in silico assay was performed, aiming to understand the reactive points of the chemical structure of RSV-GS when compared with RSV. A lower number of alerts related to reactive points were detected in the RSV-GS’ structure in comparison with RSV. Thus, RSV-GS exhibited an ameliorated profile due to the molecular modification performed and the sulfation of hydroxyl groups in RSV. The use of the in vitro approach based on the 442E OECD guideline confirmed the low sensitization potential of RSV and RSV-GS. Interestingly, RSV-GS revealed lower RFI (%) values than the parent compound RSV, concomitantly displaying the ability to mitigate the allergic reaction induced by the strong allergen DNFB in THP-1 cells. Overall, the results herein achieved reported, for the first time, the anti-allergic activity and low sensitization potential of RSV-GS, showing that the sulfonation of all the hydroxyl groups reinforces its use in cosmetic formulations. More studies should be performed to investigate the ex vivo/in vivo efficacy and evaluate the action of hydrolytic skin enzymes that locally cleave sulfate groups, metabolizing RSV-GS into the bioactive RSV compound.

## Figures and Tables

**Table 1:** In silico results for skin sensitization and skin irritation potential obtained from ADMETLab 2.0 software.

Compound	Skin Sensitization Rule	Reactive Points (Highlighted with Red)
**RSV**	Four alerts (reactive points in the structure)	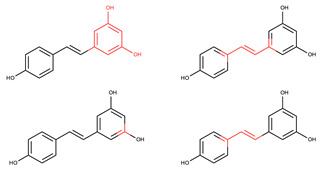
**RSV-GS**	Three alerts (reactive points in the structure)	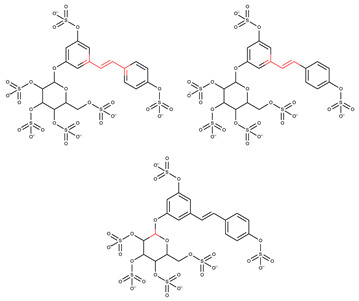

## Data Availability

Not applicable.
